# Cascade strategies for the full valorisation of Garganega white grape pomace towards bioactive extracts and bio-based materials

**DOI:** 10.1371/journal.pone.0239629

**Published:** 2020-09-18

**Authors:** Stefania Monari, Maura Ferri, Micaela Vannini, Laura Sisti, Paola Marchese, Maria Ehrnell, Epameinondas Xanthakis, Annamaria Celli, Annalisa Tassoni

**Affiliations:** 1 Department of Biological, Geological and Environmental Sciences, University of Bologna, Bologna, Italy; 2 Department of Civil, Chemical, Environmental and Materials Engineering, University of Bologna, Bologna, Italy; 3 Department of Agriculture & Food, RISE – Research Institutes of Sweden, Gothenburg, Sweden; Institute for Biological Research "S. Stanković", University of Belgrade, SERBIA

## Abstract

Agro-waste reduction and reuse are among the current main social challenges. In this perspective, the present research was aimed at the complete valorisation of Garganega grape pomace by recovering bioactive phenol extracts and by testing the solid fibre extract residues in composite formulation for packaging applications. The pomace was derived from white wine production, therefore, respect to red pomace, it was promptly removed from must after pressing, and its exploitation can be particularly interesting and valuable as still rich in active compounds. Phenol extracts were obtained both via solvent-based and pressurised liquid extractions and their phytochemical compositions were compared in terms of total amount of phenols, flavonoids, flavanols, anthocyanins, hydroxycinnamic acids, and reducing sugars. Antioxidant activity and detailed phenol profiles were also achieved. The highest phenol yield was obtained via solvent-based extraction with 75% acetone (v/v), solid/liquid ratio 1:5, 2h incubation at 50°C (77.9 gGAeq/kgDW). The fibrous solid residue of the extraction was characterized via thermogravimetric analysis and used for composite preparation by melt mixing with the renewable and biodegradable PHBV polymer through a green approach (solvent-less process). The composites resulted thermally stable at high temperatures, showing initial degradation processes only at temperatures higher than 250°C. Differential scanning calorimetry analyses were carried out to study melting and crystallization phenomena, while mechanical properties were investigated by tensile tests. The materials finally showed properties similar to those of the matrix. The bio-composites can be considered as an alternative to plain PHBV, since they are less expensive and eco-friendlier thanks to a reduced polymeric content, and they could represent a suitable way for full agro-waste exploitation.

## Introduction

Society is currently facing an ever increasing need to find new valorisation routes for agro-waste and food-industry processing residues in order to reduce their environmental impact and to implement a new management strategy for the agricultural waste in a circular bio-based economy [1]. The efficient use of agro-industrial and food by-products, residues and wastes offers a new perspective on a wide range of benefits in addition to their employment as feeds or in the biogas production. Innovative biorefinery extraction cascades may in fact allow the recovery of bioactive molecules and fibers from such poorly exploited biomass streams, thus finding applications in several industrial fields, and meeting the increasing demand from consumers for sustainable products and materials [1–3].

Grape (*Vitis* sp.) is the world’s largest fruit crop with about 74 million tons in 2017. and China is the first producer (about 17.7% of global grape production) followed by Italy (about 9.7%) and the United States (about 9.0%) [4]. Grapes are mostly used in winemaking: in the process, approximately 20% of their weight ends up as pomace [5], the primary by-product consisting in stems, skins and seeds and originated during the production of must by pressing whole grapes. Other winemaking by-products are grape stems and leaves (removed before the vinification steps to avoid an excessive astringency of the wine or a negative impact on the organoleptic characteristics), wine lees (the residues formed at the bottom of vat after fermentation or obtained after the filtration or centrifugation) and wastewater [2, 6]. All these by-products are still rich in valuable components and compounds. Therefore, the development of innovative procedures to recycle, reuse, and recover such residues is consistent with the growing demand for green materials and renewable sources of nutrients and bioactive compounds for the feed/food, pharmaceutical and cosmetic sectors, thus leading to a reduced dependence of the current manufacturing activity from the starting raw materials [2, 3, 6]. In particular, grape pomace is a low-value by-product that can be easily valorised as it is rich in several valuable compounds, such as phenolic compounds (*e*.*g*. flavonoids, phenolic acids and stilbenes [7, 8]) having ascertained biological activities beneficial to human health [2, 3, 6]. Grape pomace phytochemicals are responsible agents for multiple benefits involved in the prevention of degenerative processes through their integration into functional foods, nutraceuticals and cosmetics. Hence, the most relevant activities attributed to bioactive phytochemicals from winery by-products are antioxidant, antimicrobial, anti-inflammatory and anticancer ones [6, 9], some of these also proven *in vivo*, such as the antioxidant and anti-hypertensive effects observed in rats [10]. For this reason, grape pomace is exploitable in the food, feed, cosmetic and pharmaceutical industries [8].

Several publications reported the optimization of red and white pomace extraction techniques in order to obtain phenolic fractions showing potentially beneficial biological activities [7, 11, 12]. Most of the published studies used conventional solvent extraction for polyphenol recovery, while in recent years the need for more green technologies moved research towards other methodologies, such as pressurised liquid extraction (PLE), supercritical fluid extraction [8, 13, 14] or hydrolysis by cell wall polysaccharide degrading enzyme mixtures [11, 12]. When a specific molecule could be isolated from the mixture of extracted compounds, this was also further valorised as a building block for new bio-based polymeric structures. For example, the largely present condensed tannins could be further depolymerized to achieve gallic acid-based structures exploited as monomers for new bio-based polymers, or derivatised to produce thermoset epoxy resins [15].

Following the biorefinery approach, the exhausted grape pomaces are usually earmarked to composting or energy production plants [2, 16] but they are still exploitable to produce lasting materials in which the carbon remains sequestered, thus further reducing the environmental impact [17]. Indeed, in addition to phenolic compounds, grape pomace also contains fibres formed by a variety of polysaccharides, such as cellulose, hemicellulose and pectin, that can be exploited as a filler in polymer composites. It is in fact known that natural fibres can replace synthetic fibres in composites for specific applications [18, 19] since they are at the same time sustainable and biodegradable, easily available in nature and showing low density optimal for the production of light-weight materials. Specifically, grape pomace can fulfil the criteria identified as necessary to produce composites with attractive properties, *i*.*e*. homogeneity, sufficient availability, reasonable price, minimal need of extensive processing, biodegradability, compatibility with the polymeric matrix and polymer processing adaptability [20]. Literature reports examples of whole pomace residues inserted in poly(butylene succinate) [21], epoxy and polylactic acid matrixes [22, 23], of grape peels, seeds or stalks mixed with polypropylene [24]. The presence of low-value grape pomace fibres in a composite decreases the amount of the highly expensive polymer, thus contributing to lower the cost of the final material. Improvements in mechanical properties and matrix thermal stability were observed in composites obtained by using different types of natural fibres [15, 25].

Fully bio-based composites can be produced by using also a renewable and biodegradable matrix, such as poly(hydroxybutyrate-co-hydroxyvalerate) (PHBV) matrix, together with natural fibres. PHBV is a copolyester synthesised intracellularly by a range of bacteria from renewable/waste resources. This polymer shows biodegradability, biocompatibility and thermoplasticity, despite some noted drawbacks concerning its technical applications are known, such as a narrow processing window and low mechanical performances. Literature reports some attempts to enhance its physical and mechanical properties by blending or filling techniques, using different types of natural fibres [26–28]. Recently, the use of wine lees as filler for PHBV was described [29] demonstrating the economic feasibility of PHBV-wine lees biocomposites and reporting an economic profit ranging between 0.05 and 1.02 €/kg of composite, as a function of the filler amount.

The great potential of winemaking residues as biorefinery feedstocks was already explored by several authors [3, 30], but, in most cases, *zero waste* was not achieved or was reached only by using the final processing residues for energy production [2]. Environmental and technoeconomic aspects must also be taken into consideration. Life Cycle Assessment (LCA) is the most quoted methodology for biorefinery evaluation, as a tool to quantify the sustainability of the processes or end products [2]. LCA was successfully applied to different vine shoot biorefinery scenarios [31], while it seemed more difficult the application of LCA to the whole winemaking process, since the drafting of the inventory table for winery wastes must take into account the grape quality, production unit and many different vinification steps [2].

The ultimate aim of the present research was indeed the complete valorisation of Garganega grape pomace, a grape cultivar typically grown in the Veneto region (North-Eastern of Italy) and widely used in the production of many regional white wines, such as Prosecco, the most marketed wine worldwide. The choice of the feedstock was guided by the fact that white wine industries produce larger volumes of pomace, having a higher polyphenolic content, respect to red wine ones. In fact, the white wine vinification process involves the immediate removal of the pomace from the must, just after a soft grape pressing, without subjecting them to fermentation and thus avoiding the alcoholic extraction of their phytochemicals [3, 12]. Therefore, given the higher content of exploitable compounds, in the present research a white grape pomace was subjected to processes similar to those previously tested on red pomace [14]. A cascading approach valorisation strategy was set up to recover soluble bioactive molecules, by comparing two different extraction techniques (solvent-based and PLE), as well as to produce fully bio-based composites for packaging applications, aiming at a *zero waste* circular economy concept of the grape agro-industrial pipeline.

## Materials and methods

### Materials

White grape pomaces of *Vitis vinifera* L., Garganega cultivar, were provided by InnovEn Srl (Verona, Italy). Pomace was only softly pressed and collected right after wine production, and contained berry skins, seeds, petioles and stalks. The pomace dry weight (DW) was about 30.0% of the fresh weight (FW) and was determined by weighing aliquots of 3 gFW placed at 80°C for 48h.

The polymer used as matrix for composites was the commercial polyhydroxyalkanoate PHI 002 (NaturePlast, Ifs, France): it is the copolymer poly(3-hydroxybutyrate-co-3-hydroxyvalerate) (PHBV), containing 2 mol% of hydroxyvaleric unit and 98 mol% of hydroxybutyric unit (as determined by ^1^H NMR analysis).

### Solvent-based extraction (SE) of phenols

Garganega pomace was ground in a kitchen blender and stored at -20°C until further use. The pomace was not dried as the use of fresh feedstock would simplify the upscale of the process. Four types of solvent (ethanol, acetonitrile, acetone and methanol) were tested at three different aqueous solution concentrations (25%, 50%, 75% v/v). Each one of the 12 solvent aqueous solutions was added to sample aliquots at solid/liquid ratios (S/L) of 1:5 (5 gFW + 25 mL solvent) and 1:10 (3 gFW + 30 mL solvent), incubated at 30°C in a shaking water bath for 2h and centrifuged (5 min, 4500*g*) to separate the liquid extract from fibre residue. Different incubation temperatures (50°C, 70°C) and times (1h, 4h) were also tested. Water controls were performed in order to detect the minimum level of extractable phenols in each testing condition [32].

### Pressurised liquid extraction (PLE) of phenols

Frozen white pomace (40 gFW) was milled in a mixer (C3, Empire Sweden AB, Bromma, Sweden) for 3x15 sec at low speed allowing disruption of skins and stems and, partially, of the seeds, and stored at -40°C for further analyses.

PLE of phenols was performed using a laboratory scale SFE-500M1-2-C50 equipment (Waters, Pittsburgh, PA) system on 10.63 gFW of milled pomace into the 100 mL extraction vessel. Extraction conditions were 100 bar for 1h at 80°C with 75% EtOH-H_2_O (50:50 v/v) and 25% CO_2_, with 8 g/min total flow rate. They were based on previous study and preliminary trials [14]. Under the selected conditions of pressure and temperature the solvent mixture was at the subcritical point [33].

Extract samples were collected every 10 min and stored at -40°C until characterisation analyses. Extractions were run in duplicates.

### Liquid extracts’ spectrophotometric analysis

Garganega pomace extracts were characterised for total contents of major phenol families by spectrophotometric assays: phenols [34], flavonoids [34], flavanols [35], anthocyanins [36] and hydroxycinnamic acids [37]. The content in reducing sugars was also measured by 3,5-dinitro salicylic acid (DNS) method [38]. For each assay, a dose-response calibration curve was plotted by using a specific standard compound. The results were expressed as g of standard equivalents per kg of pomace DW. The standard compounds used were: gallic acid (GA) for phenols (0–15 μg), catechin (CAT) for flavonoids (2–14 μg) and flavanols (0–50 μg), ferulic acid (FA) for hydroxycinnamic acids (1–1000 μg), glucose (GLUC) for reducing sugars (50–500 μg). Anthocyanin results were converted from absorbance to malvidin-3-glucoside (MALV) equivalents [39].

The antioxidant activity was determined by ABTS (2,2′-Azino-bis(3-ethylbenzothiazoline-6-sulfonic acid)) assay as described by Ferri et al. [34]. The results were expressed as g of ascorbic acid (AA) equivalents per kg of pomace DW by means of a calibration curve (0–2 μg of AA).

### Characterisation of specific phenols by HPLC-DAD

Extract aliquots of 1 mL were analysed for the identification and quantification of specific phenols via HPLC-DAD. When a solvent was present, it was evaporated and replaced with water, then phenols were purified and concentrated via solid-phase extraction (Strata-X column, 33 mm polymeric sorbent 60 mg in 3 mL, Phenomenex, Torrence, CA, USA). Chromatographic analyses were performed by using a HPLC system (column Gemini C18, 5 μm particles 250 x 4.6 mm, pre-column SecurityGuard Ea, Phenomenex, Torrence CA, USA) equipped with an on-line diode array detector (MD-2010, Plus, Jasco Instruments, Großumstad, Germany), as described by Ferri et al. [36]. The adopted HPLC-DAD separation procedure allowed the simultaneous analysis of the following 28 compounds: gallic (GA), protocatechuic (PROTA), syringic (SYRA), vanillic (VANA), trans-ferulic (FERA), caffeic (CAFA), chlorogenic (CLORA), p-coumaric (CUMA), sinapic (SINA) and *trans*-cinnamic (CINA) acids, epigallocatechin (EGC), catechin (CAT), epicatechin (EC), epicatechin gallate (ECG), epigallocatechin-gallate (EGCG), vanillin (VAN), rutin (RUT), quercetin (QUERC), naringenin (NAR), myricetin (MYR), kaempferol (KAE), *trans*- and *cis*-resveratrol (tRESV, cRESV), *trans*- and *cis*-piceid (tPIC, cPIC), *trans*- and *cis*-resveratroloside (tRDE, cRDE), piceatannol (PICEAT). For each white grape pomace sample, five chromatograms obtained at different wavelengths (270, 285, 305, 323 and 365 nm) were analysed to determine the concentration of single compounds, depending on their maximum absorbance.

### Solid fibre residue characterisation

The solid residue of Garganega white pomace (WR) coming from selected best extraction process (SE 75% acetone, 50°C, 2h) underwent a thermogravimetric analysis (TGA) performed with a Perkin Elmer TGA4000 apparatus (Milan, Italy) in nitrogen (gas flow: 40 mL/min) at 10°C/min heating rate, from 30 to 700°C.

### Composite preparation and characterization

For composite preparation WR were dried at 70°C under *vacuum* for 24h, chopped up with Ika M20 Mill (Staufen, Germany) and sifted through a sieve (mesh 0.4 mm). The treated fibres and the commercial PHBV were again *vacuum* dried at 60°C overnight. The composites were prepared by melt mixing at 200°C for 5 min in a Brabender micro-compounder as previously reported [14]. Different blends were prepared with 5%, 10% and 20% (w/w) of residue and were named PHBV-5WR, PHBV-10WR and PHBV-20WR, respectively. The same thermal process was applied to a pure PHBV in order to have a reference control material.

The composites and PHBV were characterised by TGA in nitrogen (gas flow: 40 mL/min) at 10°C/min, from 30 to 700°C. The degradation temperature (T_D_) was calculated as the temperature of the maximum degradation rate, whereas the onset degradation temperature (T_onset_) was defined as the initial temperature of degradation, corresponding to the intercept of the tangent drawn at the inflection point of the decomposition step with the horizontal zero-line of the thermogravimetric curve. Calorimetric analysis was carried out with a Perkin Elmer DSC6 calorimeter (Milan, Italy), calibrated with high-purity standards. The measurements were performed under nitrogen flow as follows: first scan, from 30 to 210°C at 20°C/min and 1 min of isotherm at 210°C; cooling scan, from 210 to 0°C at 20°C/min and 1 min of isotherm; second scan, from 0 to 210°C at 20°C/min.

Molecular weights (expressed as polystyrene equivalent) were determined by gel permeation chromatography (GPC), using a GPC Agilent Series 1100 instrument equipped with a PL gel 5μ Mixed-C column (Milan, Italy) held at 30°C and Reflective Index Detector. Chloroform was used as the eluent and a calibration plot was constructed with polystyrene standards. After dissolution in a mixture of CHCl_3_/1,1,1,3,3,3-hexafluoro-2-propanol (HFIP) 95/5 (v/v), all the samples were filtered through a Teflon 0.45 μm pore size syringe filter in order to remove the insoluble lignocellulosic fraction.

Tensile properties of composites were determined on dumbbell-shaped specimens (2×5×30 mm) obtained by injection moulding (MegaTech Tecnica DueBi injection moulding machine; Ancona, Italy), working between 150 and 165°C. The tests were carried out by means of an INSTRON 5966 dynamometer (Turin, Italy) equipped with a 10 kN load cell (test speed 5 mm/min, room temperature 19 ± 1°C and 70 ± 10% of relative humidity).

### Statistical analysis

All the extractions were repeated at least twice and the experimental data were expressed as mean ± SD. All spectrophotometric assay procedures and HPLC-DAD analyses were performed in triplicate in two technical replicates each and the results are expressed as the mean (n = 3) ± SD per kilogram of dry weight (kgDW) or per litre of extract. Data were tested for normality using the Shapiro-Wilk normality test and for homogeneity using the Levene’s test for Homogeneity of Variance with default parameters from the package “car” (https://CRAN.R-project.org/package=car). The parametric one-way ANOVA test, followed by a Tuckey HDS test, were used to evaluate the differences among compared extracts. All the statistical analyses were performed using R software version 4.0.2 (R Core Team, Vienna, Austria).

## Results and discussion

### Optimization of phenol extraction

Two different methods, solvent-based (SE) and pressurised liquid extraction (PLE), were applied to Garganega grape pomace and total phenol contents were assessed (Table 1).

**Table 1:** Optimisation of solvent-based (SE) and of pressurised liquid (PLE) extraction protocols from Garganega white grape pomace.

Solvent type	Solvent (v/v)	S/L	Temp.	Time	Total phenols	
					gGAeq/L	gGAeq/kgDW
**1**^**st**^ **set of experiments: SE**						
ethanol	25%	1:10	30°C	2h	0.88 ± 0.06 ^a^	30.52 ± 2.17 ^a^
	50%				1.13 ± 0.03 ^b^	39.27 ± 1.03 ^b^
	75%				0.86 ± 0.01 ^a^	30.07 ± 0.34 ^a^
	25%	1:5			1.41 ± 0.07 ^c^	24.48 ± 1.26 ^c^
	50%				1.81 ± 0.01 ^a^	31.60 ± 0.15 ^a^
	75%				1.34 ± 0.10 ^c^	23.33 ± 1.55 ^c^
acetonitrile	25%	1:10			1.36 ± 0.05 ^d^	47.53 ± 1.68 ^d^
	50%				1.56 ± 0.03 ^e^	54.32 ± 0.94 ^e^
	75%				1.17 ± 0.18 ^b^	40.80 ± 6.26 ^b^
	25%	1:5			2.29 ± 0.05 ^b^	39.81 ± 0.86 ^b^
	50%				3.93 ± 0.23 ^f^	68.47 ± 3.94 ^f^
	75%				2.97 ± 0.13 ^d^	51.74 ± 2.22 ^d^
acetone	25%	1:10			1.43 ± 0.02 ^d^	49.93 ± 0.64 ^d^
	50%				1.61 ± 0.06 ^e^	56.24 ± 2.07 ^e^
	75%				1.67 ± 0.06 ^e^	58.22 ± 2.12 ^e^
	25%	1:5			3.41 ± 0.15 ^e^	59.32 ± 2.59 ^e^
	50%				3.94 ± 0.15 ^f^	68.64 ± 2.71 ^f^
	75%				4.21 ± 0.23 ^f,i^	73.34 ± 3.94 ^f,i^
methanol	25%	1:10			0.45 ± 0.02 ^g^	15.68 ± 0.59 ^g^
	50%				0.85 ± 0.01 ^a^	29.53 ± 0.17 ^a^
	75%				0.76 ± 0.03 ^a,c^	26.34 ± 1.13 ^a,c^
	25%	1:5			1.92 ± 0.05 ^a^	33.36 ± 0.86 ^a^
	50%				2.77 ± 0.20 ^d^	48.17 ± 3.33 ^d^
	75%				2.38 ± 0.02 ^b^	41.38 ± 0.37 ^b^
water	100%	1:10			0.41 ± 0.00 ^g^	14.43 ± 0.05 ^g^
		1:5			0.60 ± 0.01 ^h^	10.50 ± 0.18 ^h^
**2**^**nd**^ **set of experiments: SE**						
acetonitrile	50%	1:5	50°C	1h	3.75 ± 0.02 ^f^	65.24 ± 0.37 ^f^
				2h	**3.48 ± 0.04** ^**e,f**^	**60.63 ± 7.39** ^**e,f**^
				4h	3.47 ± 0.02 ^e^	60.37 ± 0.37 ^e^
acetone	75%			1h	3.71 ± 0.06 ^f^	64.63 ± 0.99 ^f^
				2h	**4.47 ± 0.07** ^**i**^	**77.87 ± 1.23** ^**i**^
				4h	4.11 ± 0.04 ^f^	71.60 ± 0.74 ^f^
methanol	50%			1h	2.26 ± 0.08 ^b^	39.37 ± 1.48 ^b^
				2h	**2.22 ± 0.03** ^**b**^	**38.59 ± 0.62** ^**b**^
				4h	2.70 ± 0.01 ^d^	46.95 ± 0.12 ^d^
water	100%			1h	1.02 ± 0.05 ^g^	17.75 ± 0.86 ^g^
				2h	**0.95 ± 0.02** ^**g**^	**16.55 ± 0.34** ^**g**^
				4h	0.85 ± 0.02 ^g^	14.86 ± 0.32 ^g^
acetonitrile	50%	1:5	70°C	1h	3.69 ± 0.02 ^f^	64.20 ± 0.37 ^f^
				2h	3.63 ± 0.15 ^f^	63.15 ± 2.59 ^f^
				4h	4.22 ± 0.08 ^f,i^	73.52 ± 1.48 ^f,i^
acetone	75%			1h	3.77 ± 0.06 ^f^	65.68 ± 0.99 ^f^
				2h	4.01 ± 0.06 ^f^	69.86 ± 0.99 ^f^
				4h	3.88 ± 0.02 ^f^	67.51 ± 0.37 ^f^
methanol	50%			1h	2.83 ± 0.02 ^d^	49.22 ± 0.37 ^d^
				2h	3.14 ± 0.04 ^e^	54.70 ± 0.74 ^e^
				4h	3.50 ± 0.00 ^e^	60.98 ± 0.00 ^e^
water	100%			1h	1.22 ± 0.02 ^c,g^	21.27 ± 0.42 ^c,g^
				2h	1.36 ± 0.03 ^c^	23.66 ± 0.49 ^c^
				4h	1.37 ± 0.00 ^c^	23.92 ± 0.07 ^c^
**3**^**rd**^ **set of experiments: PLE**						
ethanol	50%	1:126*	80°C	30 min	**1.07 ± 0.19** ^b,d^	**40.27 ± 6.26** ^b,d^
		1:196*		40 min	**0.87 ± 0.07** ^d,e^	**51.09 ± 4.20** ^d,e^
		1:256*		50 min	**0.64 ± 0.07** ^d^	**48.98 ± 5.09** ^d^
		1:333*		60 min	**0.61 ± 0.06** ^e,f^	**60.78 ± 6.19** ^e,f^

Total phenols were quantified and data expressed as g of GA equivalent per litre of extract (gGAeq/L) and as g of gallic acid (GA) equivalent per kg of pomace dry weight (gGAeq/kgDW). Different letters indicate statistically significant differences (one-way ANOVA followed by a followed by a Tuckey HDS test, *p* < 0.05) among data expressed with the same measure unit along the same column. Data are the mean ± SD (n = 3). In bold, the treatments selected for further characterization analyses. S/L, solid/liquid ratio (kgFW of pomace/L of solvent).

* PLE is a continuous process where the mobile phase of the solvent mixture flows through the working material which is stationary, therefore the S/L ratio is different according to the time of sampling.

In a first set of experiments, SE methodology was applied by using four different solvents (ethanol, acetonitrile, acetone and methanol) chosen among the most studied solvents for polyphenol extraction [40], at 3 different concentrations (25, 50, 75% v/v) and two solid/liquid ratios (S/L; 1:10 and 1:5), incubation at 30°C for 2h. Water controls were also performed.

All solvent extracts showed higher amounts of phenols with respect to water control. The maximum yield was obtained by using 75% (v/v) acetone and S/L 1:5 (4.2 gGAeq/L, equivalent to 73.3 gGAeq/kgDW, Table 1). S/L 1:5 showed an average increase of 2.7-times as compared to the level of phenols per L of extract released by solvents (1.4-times when data are given per kgDW), with the only exception of ethanol. Extractions with 50% (v/v) solvent concentration showed the highest amount of phenols (on average 5.2-fold and 3.1-fold higher than water respectively for 1:5 and 1:10 S/L ratios), with the exception of acetone that was more efficient at 75% (v/v) concentration (7.0-fold and 4.0-fold higher than control for 1:5 and 1:10 S/L respectively) (Table 1). Reported data showed a higher phenol yield as compared to previous published papers on grape pomace in which more concentrated solvents or more complex mixtures were used, such as 80% ethanol [41], acetone:water:acetic acid (90:9.5:0.5) or methanol:water:acetic acid (90:9.5:0.5) [42]. A similar extraction process applied to red pomace (Merlot cultivar) also gave 40% lower yield (up to 47 gGAeq/kgDW) [14]. Solvent concentrations higher or lower than the 50–75% range used (Table 1) were found to reduce phenol extraction efficiency in both white and red pomace [32, 43]. The most efficient S/L in terms of total phenol yield resulted to be 1:5, in accordance with some literature data [43]. The reduction of solvent consumption, by using 1:5 S/L, might yield both environmental and economic advantages, due to lower pollution and costs in case of industrial scale-up processing. Moreover, a potential solvent recovery can be evaluated in industrial plants by adding a final evaporation step, with the double result of solvent reuse and dried phenolic product (best-use form for storage and shipping).

In a second set of experiments, SE extraction was performed at 1:5 S/L with the three best performing solvents (50% acetonitrile, 75% acetone and 50% methanol), at different incubation temperatures (50°C and 70°C) and times (1h, 2h, 4h). 75% acetone in a 2h process proved to be the most efficient solvent at both incubation temperatures, extracting the highest yield of phenols (4.7 and 3.0-fold higher than water control for 50°C and 70°C incubations, Table 1). In 50°C processes, 75% acetone sample the phenol yield was slightly increased as compared to 30°C, reaching up to 4.5 gGAeq/L, equivalent to 77.9 gGAeq/kgDW (+58% respect to 30°C process). Instead, the increase of the incubation temperature up to 70°C did not lead to higher phenol recovery with the only exception of water control (+125% from 30°C to 70°C), while solvent extraction did not seem to be significantly affected (Table 1). To the best of our knowledge, there are no data in literature on phenol extraction from Garganega pomace. The yields here obtained were higher compared to those reported for other white grape pomace extracts with the same solvent type, *e*.*g*. up to 2.5 and 6.8-fold higher than levels reported for Parellada and Müller Thurgau pomace respectively [44, 45].

As to the effect of increased incubation time (up to 4h) on phenol extraction, the data did not indicate a univocal trend. Previous data, obtained by a combined enzymatic plus ethanol extraction both on red and white grape pomace, indicated a decrease in the amount of phenols with the increase of incubation time [11, 12]. In order to reduce processing energy costs, incubation temperature and time are major factors during the extraction optimisation [46]. Therefore, 50°C for 2 hours seemed to be the right balance between processing energy cost and high phenol recovery.

A third set of experiments was carried out by means of pressurised liquid extraction (PLE) during which samples were collected every 10 min and their total phenol content quantified (Table 1). Maximum yield was obtained after 60 min (0.61 gGAeq/L, equivalent to 60.8 gGAeq/kgDW). This content was 22% lower than the maximum yield obtained via SE (sample 75% acetone, 50°C, 2h, Table 1) when data were expressed as gGAeq/kgDW.

When comparing SE and PLE extractions performed with the same solvent, 50% ethanol, PLE gave 1.4-folds higher phenol yield with respect to SE (Table 1). PLE gained interest in recent years as it is considered as a more environmental-friendly technology in order to achieve high yields of bioactive compounds from natural sources with the use of food grade and non-toxic solvents [8]. On the other hand, S/L data reported in Table 1 proved that PLE requires large solvent amounts, being a continuous process where the mobile phase of the solvent mixture flows through the working material stationary phase. This explains the increasing phenol contents extracted over time up to the plateau reached after 60 min (data as gGAeq/kgDW, Table 1), and also the progressive decrease over time of the phenol concentration in the extract (data as gGAeq/L).

The three solvent-based extracts showing the highest content of total phenols (50% methanol and acetonitrile, 75% acetone, S/L 1:5, 50°C, 2h) plus water control, and the four PLE samples were subjected to further biochemical characterisation.

### Spectrophotometric characterisation of the extracts

**Fig 1:**
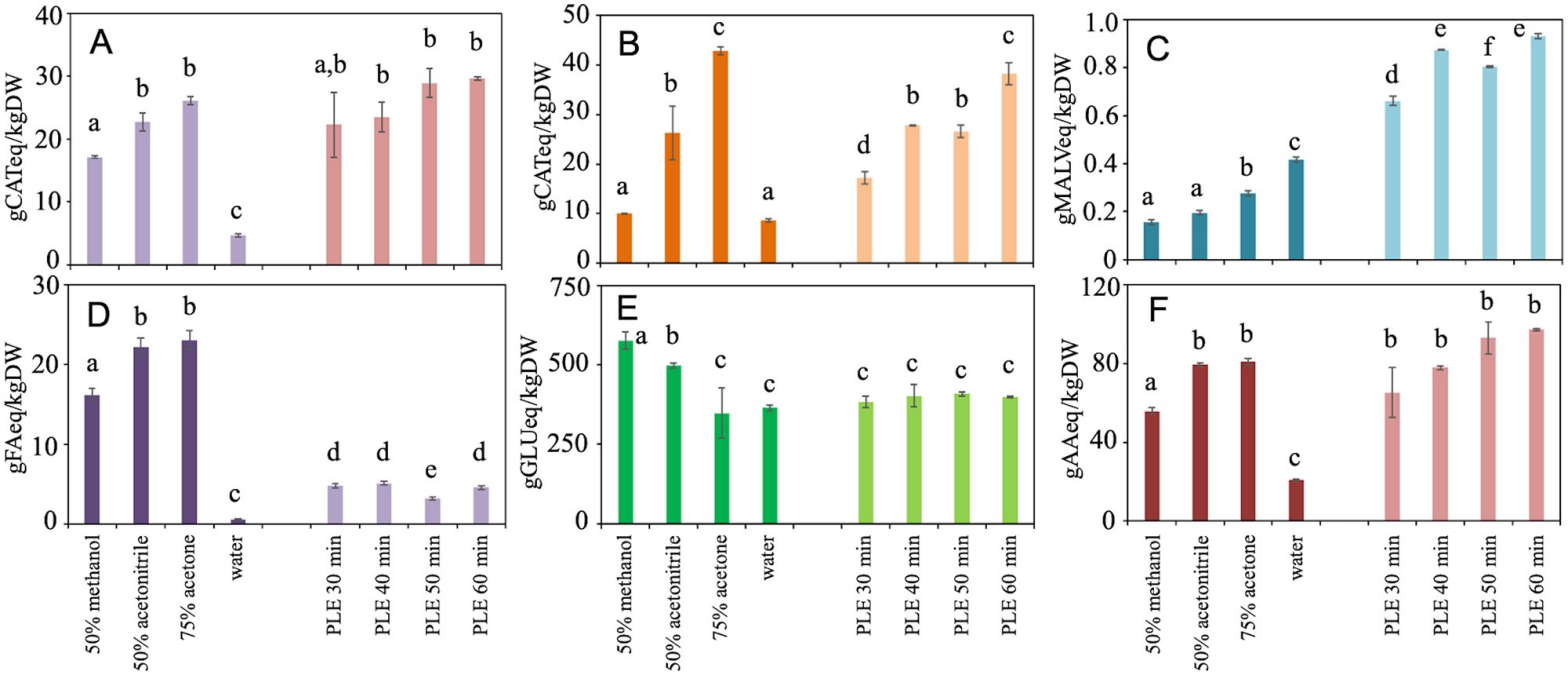
Total amounts of flavonoids (A), flavanols (B), anthocyanins (C), hydroxycinnamic acids (D), reducing sugars (E) and antioxidant activity (F) of Garganega pomace extracts. Results are expressed as g of standard compound equivalent per kg of pomace dry weight (g eq/kgDW). Different letters indicate a statistically significant difference (oneway ANOVA followed by a followed by a Tuckey HDS test, *p* < 0.05) between the same type of data. Data are the mean ± SD (n = 3). AA, ascorbic acid; CAT, catechin; FA, ferulic acid; GLUC, glucose; MALV, malvidin.

Spectrophotometric techniques were used to characterise the phytochemical profiles and antioxidant activity of the previously selected SE and PLE samples (Fig 1). In addition to total phenols previously reported in Table 1, the content of the most relevant phenolic compound families was determined (Fig 1A–1D). Reducing sugars levels (Fig 1E) and total antioxidant activity (Fig 1F) were also evaluated.

The content in flavonoids was similar in most SE and in all PLE samples (on average 22.5 gCATeq/kgDW) being 3-5-fold higher than SE water control (Fig 1A). The present data seemed to be consistent with total flavonoid concentrations previously detected in white grape pomace treated with different solvents such as Chardonnay (80% ethanol, S/L 1:10; *e*.*g*. 16.2 gRUT/kgDW) [41] or Riesling (several ethanol and methanol extractions, S/L 1:10; *e*.*g*. 0.2–5.4 gQUERC/kgFW) [47]. Flavanols were almost equally extracted by SE 75% acetone (5-fold higher than control) or by 60 min PLE processes (on average 40.6 gCATeq/kgDW) (Fig 1B) and values of the same magnitude than those reported for Müller Thurgau and Morio Muscat cultivars (25.0–58.9 gCAT/kgDW) [44]. As expected, being a white cultivar, Garganega anthocyanin contents were low even if PLE methodology was able to extract 3.8-fold higher contents (average of 0.82 gMALVeq/kgDW) than SE (Fig 1C). On the contrary, the selected solvents resulted more efficient than PLE in extracting hydroxycinnamic acids from Garganega pomace (Fig 1D) and the highest yields detected with 75% acetone and 50% acetonitrile (on average 22.6 gFAeq/kgDW), 5.1-fold higher than the average of PLE samples (Fig 1D).

Reducing sugars were mainly extracted by 50% methanol and 50% acetonitrile (577.2 and 497.6 gGLUeq/kgDW respectively), while 75% acetone and PLE extracts showed levels similar to water (on average 384.6 gGLUeq/kgDW) (Fig 1E). These results were consistent with those obtained by similar processes with Merlot cultivar (up to 209 and 302 gGLUeq/kgDW by SE and PLE respectively) [14].

All the extracts exerted in fact an *in vitro* antioxidant activity (Fig 1F). The most active SE extracts were those obtained with 50% acetonitrile and 75% acetone (average of 80.2 gAAeq/kgDW, 4-fold higher than water control). In PLE, the antioxidant capacity slightly increased with the extraction time (50–60 min, average of 95.2 gAAeq/kgDW). The detected bioactivity levels were similar to those reported by Gonzàlez-Centeno et al. [45] (ABTS assay, 72–134 gTrolox/kgDW) or 6-9-folds higher than Deng et al. [44] results (DPPH assay, 11–16 gAAeq/kgDW) on different white grape pomace extracts.

In summary, among the analysed SE extracts, 75% acetone was able to recover the highest levels of total flavonoids, flavanols, hydroxycinnamic acids and anthocyanins (Fig 1A–1D), while reducing sugars were more soluble in 50% methanol (Fig 1E). PLE extracts had a similar composition over time and showed levels of flavonoids, flavanols and sugars comparable to those of 75% acetone SE samples. All the analysed metabolite families were detected at levels generally higher or of the same order of magnitude than those already reported for white pomace solvent extracts of different cultivars [41, 44, 47].

### HPLC-DAD analysis of specific phenols

**Fig 2:**
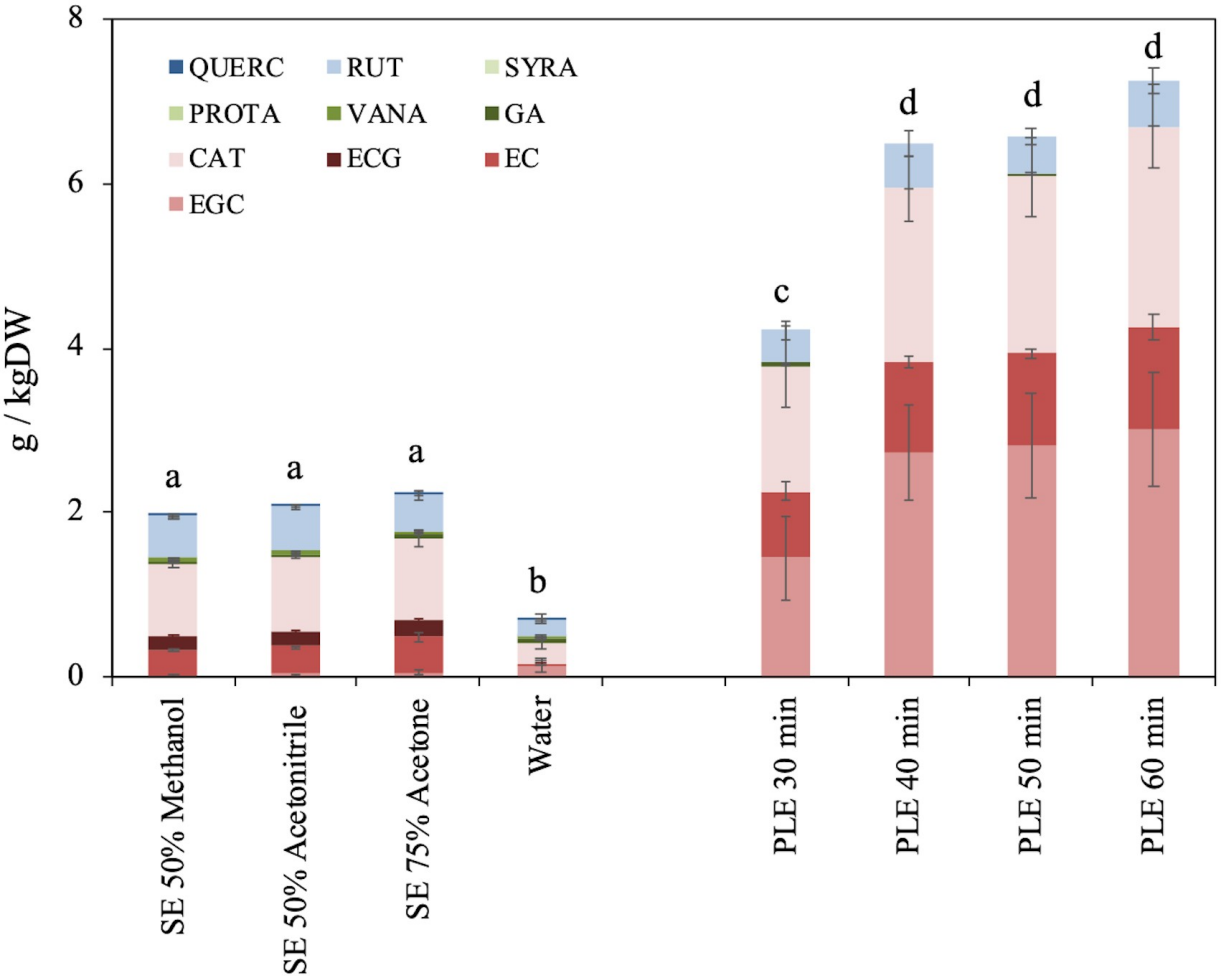
HPLC-DAD characterization of phenols extracted from Garganega pomace. Results are expressed as g of standard compound equivalent per kg of pomace dry weight (g/kgDW). Different letters indicate a statistically significant difference (oneway ANOVA followed by a followed by a Tuckey HDS test, *p* < 0.05) between the same type of data. Data are the mean ± SD (n = 3). CAT, catechin; EC; epicatechin; ECG, epicatechin gallate; EGC, epigallocatechin; GA, gallic acid; PROTA, protocatechuic acid; QUERC, quercetin; RUT, rutin; SYRA, syringic acid; VANA vanillic acid.

The phenolic profile of Garganega pomace extracts was determined by HPLC-DAD (Fig 2). Twelve different compounds were detected in SE extracts, belonging to three different classes of phenols: flavonols (QUERC, RUT), hydroxybenzoic acids (SYRA, PROTA, VANA, GA) and flavanols (CAT, ECG, EC, EGC). Overall, flavanols proved to be the largest class of identified compounds (about 71% of the total amount), followed by flavonols (24%). On average, SE samples showed about 2.1 g/kgDW of total identified phenols (3-fold higher than in water sample).

PLE extracts contained higher levels but a lower range of identified phenol compounds with respect to SE extracts (*e*.*g*. up to 56.8-times higher EGC than the average of SE samples) (Fig 2). Most of the quantified compounds were flavanols (CAT, EC, EGC; on average the 91.5% of total detected phenols); low RUT and trace GA amounts were also detected. Previous studies about white grape winemaking by-products also reported the presence of CAT and EC as the compounds mostly present in several cultivars extracted both via SE and PLE [12, 47, 48]. Unlike the present data (Fig 2), other studies have analysed but not detected EGC and EGCG in white pomace [47, 48].

As previously stated, flavonols also abounded in Garganega extracts, with RUT being the major representative in both SE and PLE samples, while its aglycone QUERC was only recovered in SE and in low amount (Fig 2). Previous published data on different white grape varieties, also reported the presence of other flavonols such as kaempferol [47], not detected in the present study. Similarly, stilbenes were not identified in Garganega extracts (Fig 2), while they were present in Riesling [47] and in Trebbiano and Verdicchio [12] white pomace, resveratrol and piceid and resveratroloside were respectively detected. Hydroxycinnamic acids (*e*.*g*. GA and PROTA) were commonly quantified in white grape pomace extracts [12, 47, 48] consistently with Garganega results (Fig 2).

Both SE and PLE produced extracts featuring very complex molecule mixtures, with different composition depending on the applied process (Figs 1 and 2). Range and concentration of extracted phytochemicals can be ascribed to specific compound solubility in different solvents and to the diverse processing conditions.

### Composites preparation and characterisation

**Fig 3:**
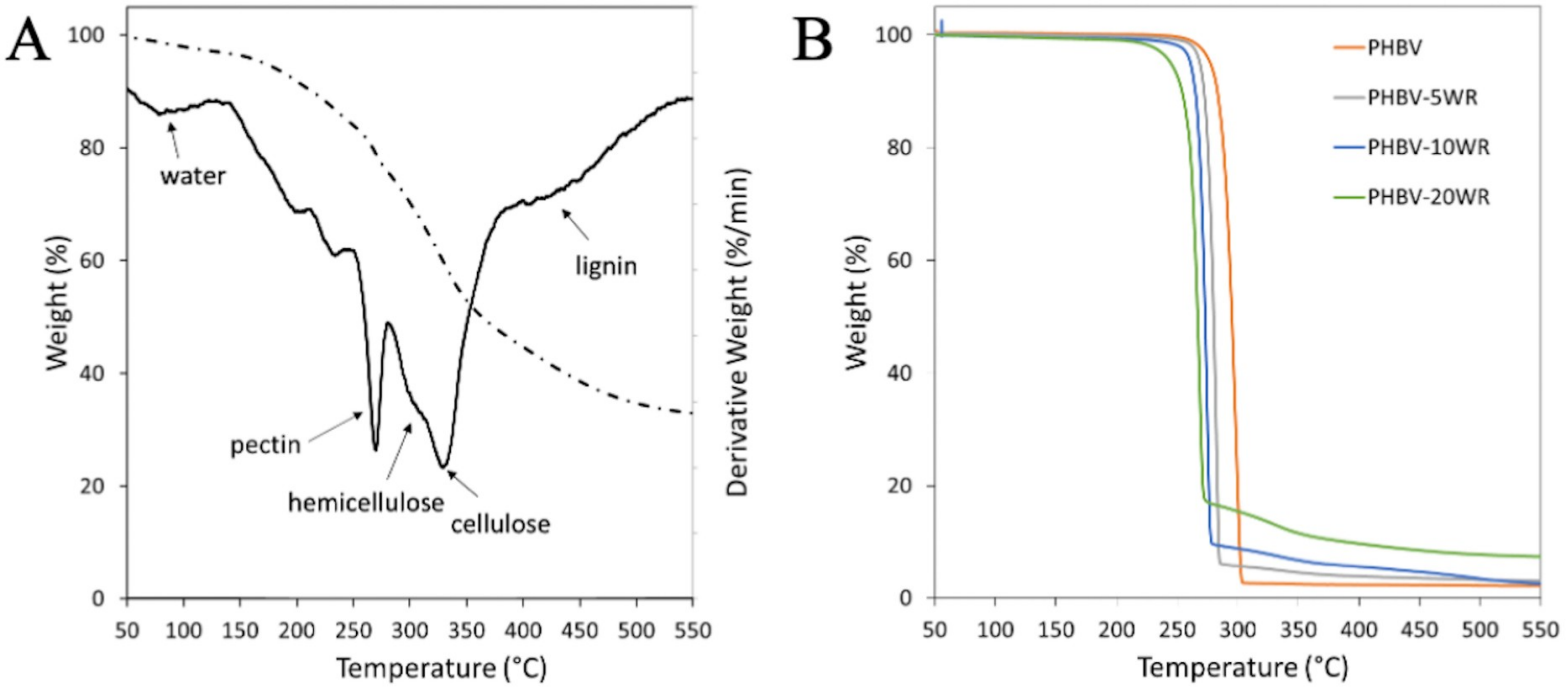
Thermogravimetric (TGA) curve (dotted line, left axis) and related derivative curve (solid line, right axis) of WR solid residue (A) and of PHBV polymer and related bio-composites including different WR percentages (B). WR was obtained after SE extraction with 75% acetone, 50°C, 2h.

The white pomace solid residue (WR), obtained after SE extraction with 75% acetone (S/L 1:5, 2h, 50°C), was characterised by thermogravimetric analysis (TGA) (Fig 3A). Considering the WR derivative trace, the WR degradation process consisted in multiple degradation steps occurring between 150 and 500°C, that could be related to the different fractions of the lignocellulosic residue [49]. The degradation steps located at around 270°C and 330°C can be ascribed to pectin, sugar, hemicellulose and cellulose components of the residues, respectively. Lignin degrades at high temperature, around 400°C. The thermogram is also characterized by a noticeable non-volatile residue that is almost exclusively due to the formation of highly condensed aromatic structures (Fig 3A).

**Fig 4:**
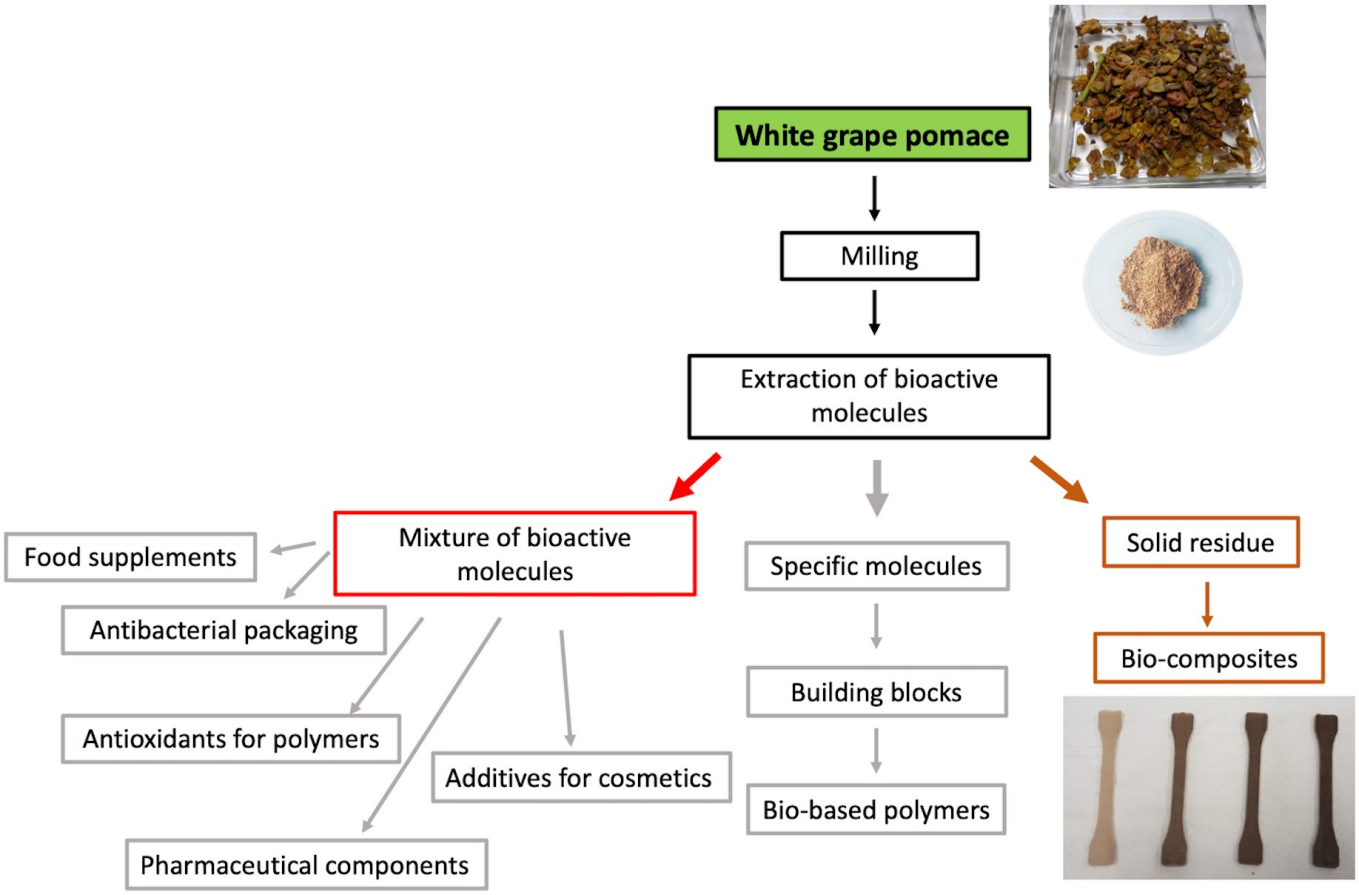
Cascading approach to fully valorise white grape pomace. In grey the applications and materials not subject of the present study.

Three bio-composites were prepared by adding 5, 10 and 20% (w/w) of WR to PHBV matrix. The filler introduction into polymer was performed by a simple melt mixing of the components at 200°C. Such a method is rapid and green, as it only requires 5 minutes of mixing and needs no solvent. The chosen mixing temperature is high enough to allow the PHBV matrix to melt and sufficiently low to protect WR from fibre degradation, as indicated by TGA analysis. As expected, the wine wastes caused a significant browning effect on the PHBV original colour (see Fig 4 in Conclusions).

The chemical, thermal and mechanical characterizations of the pure PHBV reference material and of the bio-composites are reported in Table 2. Gel permeation chromatography (GPC) analyses showed a slightly lower molecular weight (M_n_ and M_w_) of the composites with respect to the PHBV subjected to the same thermal treatment of composites. This result indicates that during processing the fillers probably formed some degradation products (water, alcoholic and carboxylic compounds) which may favour the matrix hydrolysis, as already observed by other authors [50–52].

**Table 2:** Chemical, thermal and mechanical characteristics of PHBV reference material and bio-composites.

Sample code	M_n_ ^a^	M_w_ ^a^	T_onset_	T_D_	T_m_	ΔH_m_	T_c_	ΔH_c_	T_m_	ΔH_m_	E	σ	ε
	^.^ 10^−3^	^.^ 10^−3^	(°C) ^b^	(°C) ^b^	(°C) ^c^	(J/g) ^c^	(°C) ^d^	(J/g) ^d^	(°C) ^e^	(J/g) ^e^	(MPa) ^f^	(MPa) ^f^	(%) ^f^
**PHBV**	64.7	133.2	288	302	172	78	114	73	168	82	1728 ± 50	33.2 ± 1.0	3.1 ± 0.1
**PHBV-5WR**	59.7	125.9	275	282	171	78	111	72	168	80	1638 ± 58	28.9 ± 1.3	2.9 ± 0.2
**PHBV-10WR**	60.4	132.9	268	274	170	71	111	67	168	78	1657 ± 51	27.6 ± 1.1	2.9 ± 0.1
**PHBV-20WR**	59.1	127.7	259	269	169	63	109	57	167	66	1640 ± 44	24.1 ± 0.9	2.5 ± 0.2

The sample code indicates the type of polymer (PHBV) and the percentage of white grape pomace residues (WR). M_n_, number average molecular weight; M_w_, weight average molecular weight; T_onset_, temperature of initial decomposition; T_D_, temperature of the maximum degradation rate; T_m_, melting temperature; ΔH_m_, melting enthalpy; T_c_, crystallization temperature; ΔH_c_, crystallization enthalpy; E, elastic modulus; σ, tensile strength; ε, elongation at break.

^a^ determined by gel permeation chromatography (GPC) analyses.

^b^ determined by thermogravimetric analysis (TGA) under N_2_ flux, by heating at 10°C/min.

^c^ determined by DSC during the first heating scan at 20°C/min.

^d^ determined by differential scanning calorimetry (DSC) during the cooling scan at 20°C/min.

^e^ determined by DSC during the second heating scan at 20°C/min.

^f^ determined by tensile tests.

The TGA curve of pure PHBV sample showed a single degradation step and the weight loss was completed just above 300°C (Fig 3B). Indeed, the accepted mechanism for PHB degradation consists in a random breakage of chain ester bonds to oligomers through a single step [53, 54]. On the other hand, all bio-composites presented a second degradation step starting at about 270°C, due to the filler decomposition, the duration and size of which was due to the filler content (Fig 3B). The presence of the filler decreased both the initial decomposition temperature (T_onset_) and the maximum degradation rate (T_D_) of the composites when compared to the PHBV sample. The decrement strictly depends on the WR amount, proving that the extracted fibres acted as pro-degrading agent due to their high content of hemicellulose, as previously reported for few PHB composites [53, 55, 56]. This can be confirmed also by the previously discussed GPC analysis (Table 2).

PHBV is a semi-crystalline polymer characterised by a high level of crystallinity, as confirmed by the DSC data (Table 2) that showed a particularly high melting enthalpy both in the first and the second heating scans (ΔH_m_: 78 and 82 J/g, respectively). Moreover, the polymer showed a high crystallization capability and crystallised completely during the cooling step.

DSC data of bio-composites showed that the melting temperatures (T_m_) and enthalpies (ΔH_m_) were not affected by the presence of the fillers, indicating that the crystalline phase of PHBV did not change in terms of crystal perfection and degree of crystallinity (Table 2). On the other hand, a slight reduction in the crystallization temperature (T_c_) from the melt was recorded for the composites. More specifically, considering the T_c_ decrement from 114 to 109°C, it was possible to speculate that the slight reduction in the crystallization rate could be induced by constraints on the chain mobility due to the presence of fibres. In case of highly crystalline materials like PHBV, such result is thoroughly desirable, as it allows to enlarge the processing window.

As regards the tensile properties, by comparing the reference PHBV and the related bio-composites, it was observed that Young’s modulus (E) remained fairly constant while the tensile strength (σ) and elongation (ε) at break slightly decreased. Fillers usually produce an increment in the elastic modulus of the polymer matrix [53, 55–57]. In few cases, this enhancement was not recorded [51] and such result was explained by considering that, if the elastic modulus of matrix and fibre is similar, the filler’s reinforcing effect might not be evident. The decrement in strength and elongation for composites obtained with natural fibres was quite common [51, 55, 57] and could be ascribable to a poor interfacial adhesion between hydrophobic matrix and hydrophilic filler, that involved a no efficient stress transfer between the two phases. Similar results were obtained by using red pomace residues [14], even if the composition of the fibres appeared slightly different, mainly in terms of cellulose content. In order to improve the compatibility between hydrophobic polymers and hydrophilic fibres, many chemical and physical surface treatments can be applied [58, 59] or a third component acting as a coupling agent can be added to achieve high-performance fibre reinforce composites. Therefore, new strategies can be evaluated to increase the performances of the new composites. However, as the loss in mechanical properties was not very high, and bio-composites still presented properties suitable for practical applications, the suitability of the WR residue as an efficient filler for the PHBV matrix, similarly to other types of agro-waste [60].

## Conclusions

Garganega grape pomace was subjected to a two-step cascading process aiming at a full *zero waste* valorisation of this by-product. In the first step the extraction of valuable bioactive phenols was achieved and two extraction methods, solvent-based (SE) and pressurised-liquid (PLE), were compared, and resulted both suitable for the treatment of Garganega pomace with highest total phenol recovery obtained by means of 75% acetone SE extraction. Both SE and PLE extracts showed antioxidant activity. They were very complex mixtures containing several types of metabolites, and their composition depended on the applied methodology. In view of an industrial scale up of this by-product valorisation, the selection of the best treatment could depend on the targeted compounds as well as on the environmental and economic impact of the extraction process.

Taking their composition and antioxidant activity into consideration, the recovered phenol extracts could find several different applications *e*.*g*. as food supplements, bioactive pharmaceutical components, additives for cosmetics and pharmaceuticals (Fig 4). Furthemore, these natural phytochemicals can be exploited to protect packaging polymers from oxidation as well as to produce packaging active used to extend food shelf-life. In the case of a possible isolation of a specific molecule from the extract mixture, this could also be further valorised as building block for the synthesis of novel fully bio-based polymeric structures (Fig 4).

Following phenols recovery, the solid fibre extraction residue was applied, in a content variable between 5 and 20% (w/w) to produce fully bio-based composite formulations by melt mixing together with the renewable and biodegradable PHBV matrix (Fig 4). The composites showed thermal and mechanical properties that were only slightly modified with respect to pure PHBV, despite the presence of a significant percentage of grape fibres. Therefore, solid pomace residue did not act as a reinforcing agent but as a filler without causing any processing issues and with the added-value of being a low-cost biodegradable component obtained from no-food competition biomass. For such reasons, the preparation of bio-composites can be considered as a valid approach to fully exploit agro-wastes.

In conclusion, the present study successfully demonstrated the feasibility of a double step for a 100% valorisation of grape pomace by-product. In future perspective, the great potential of such winemaking residues as biorefinery feedstock must also be evaluated from the environmental and technoeconomic points of view, by using LCA or other tools.
